# Cytokine-Based Generation of CD49a^+^Eomes^−/+^ Natural Killer Cell Subsets

**DOI:** 10.3389/fimmu.2018.02126

**Published:** 2018-09-25

**Authors:** Xiang Ni, Binqing Fu, Jinghe Zhang, Rui Sun, Zhigang Tian, Haiming Wei

**Affiliations:** ^1^Institute of Immunology and the CAS Key Laboratory of Innate Immunity and Chronic Disease, School of Life Science and Medical Center, University of Science and Technology of China, Hefei, China; ^2^Hefei National Laboratory for Physical Sciences at Microscale, University of Science and Technology of China, Hefei, China

**Keywords:** tissue-resident NK cell, cell development, interleukine-4, Eomes, CD49a

## Abstract

Recent studies have identified CD49a^+^Eomes^−^ and CD49a^+^Eomes^+^ subsets of tissue-resident NK (trNK) cells in different organs of the mouse. However, the characteristics of CD49a^+^Eomes^−/+^ NK cell development and the regulation of Eomes expression in NK cells remain unclear. Here, we established an *in vitro* cytokine-based feeder-free system in which bone marrow progenitor cells differentiate into CD49a^+^ NK cells. IL-15 was identified as being the key cytokine in this system that supported the development and maintenance of CD49a^+^ NK cells. The CD49a^+^ NK cells generated were Eomes^−^CD49b^−^ and shared the same phenotype as hepatic trNK cells. IL-4 induced the expression of Eomes in generated NK cells and converted them into CD49a^+^Eomes^+^ cells, which were phenotypically and functionally similar to uterine trNK cells. Moreover, the IL-4/STAT6 axis was identified as being important in the generation of CD49a^+^Eomes^+^ induced NK cells. Collectively, these studies describe an approach to generate CD49a^+^Eomes^−/+^ subsets of NK cells and demonstrate important roles for IL-15 and IL-4 in the differentiation of these cells. These findings have potential for developmental research underlying the generation of different subsets of NK cells and the application of adoptive NK cell transfer therapies.

## Introduction

Over recent years, researchers have shown increasing levels of interest in CD49a^+^ tissue-resident natural killer (trNK) cells (also referred to as a group of innate lymphoid cells, ILC1s), which have been found in a range of organs such as the liver, uterus, skin and salivary glands (SG) (1–6).

These trNK cells not only function to counter potential invading organisms (7), similar to conventional NK (cNK) cells, but also contribute to multiple biological processes. Murine hepatic trNK cells are thought to be involved in the immunological memory response in NK-mediated contact hypersensitivity (1). In addition, uterine NK cells can promote angiogenesis, fetal growth and immune homeostasis during early pregnancy (8–10). TrNK cells and cNK cells express a range of similar markers, such as NK1.1, NKp46, NKG2D, and T-bet, but can be distinguished by the high expression levels of CD49a, CD69, and TNF-related apoptosis-inducing ligand (TRAIL) by trNK cells; cNK cells are CD49b^+^ (2, 11, 12).

Hepatic trNK cells differ from cNK cells due to their lack of Eomesodermin (Eomes) expression. In contrast, CD3^−^NK1.1^+^CD49a^+^ cells in the uterus and SG express high levels of Eomes (2, 5, 13, 14). Evidence suggests that the homeostasis of hepatic trNK cells is disrupted by the ectopic expression of Eomes and T-bet (15). Compared to uterine NK1.1^+^CD49a^+^Eomes^−^ and cNK cell subsets, uterine NK1.1^+^CD49a^+^Eomes^+^ cells exhibit the strongest capability to secrete growth-promoting factors. However, both cell number and factor-secreting abilities are significantly reduced in *Nfil3*^−/−^ (E4BP4-deficient) mice resulting in fetal growth restriction (10). However, the regulation of Eomes expression in trNK cells has not yet been elucidated. The expression of Eomes is thought to depend upon NFIL3 (E4BP4), which promotes transcription of the Eomes gene by binding to its regulatory regions (16). Although significantly reduced in cell number, some NK cells are still known to be localized in the uterus and SG of *Nfil3*^−/−^ mice, thus suggesting an alternative molecular mechanism for the induction of Eomes (13, 17).

Existing methods for obtaining trNK cells are limited due to low cell numbers and complex tissue separation processes. Consequently, an *in vitro* generation system for CD49a^+^Eomes^−/+^ NK cells would represent a highly useful tool with which to carry out developmental and functional research, as well as facilitate the development of therapeutic applications. Research has shown that when cultured with stromal cells and cytokines, progenitor cells from bone marrow (BM), or fetal liver, can differentiate into all ILC subsets with no T or B cells (18, 19). However, it is not yet clear as to how it might be possible to differentiate progenitor cells selectively into CD49a^+^ or CD49a^+^Eomes^+^ NK-like cells.

Here, we describe the development of an *in vitro* system in which BM cells can successfully differentiate into CD49a^+^Eomes^−^ NK cells with a high proportion. In this feeder-free system, interleukin-15 (IL-15) was identified as being the key cytokine that supported the development and maintenance of these cytokine-induced NK (referred as induced NK) cells. The CD49a^+^ induced NK cells generated were Eomes^−^CD49b^−^ and shared similar phenotypes to hepatic trNK cells. Furthermore, IL-4 stimulation drove the expression of Eomes on induced NK cells, making these cells phenotypically and functionally similar to uterine NK1.1^+^CD49a^+^Eomes^+^ cells. Finally, the IL-4/STAT6 axis was identified as being important for the development of CD49a^+^Eomes^+^ induced NK cells.

## Materials and methods

### Mice

C57BL6 (B6) mice were purchased from the Shanghai Experimental Animal Center of the Chinese Academy of Science (Shanghai, China). *Nfil3*^−/−^ mice were a gift from Professor Tak Wah Mak (University of Toronto, Canada). *Tbx21*^−/−^ mice were purchased from the Jackson Laboratory. *Il4ra*^−/−^ mice were generated using CRISPR-Cas9 technology as described previously (10). All mice were bred and maintained under specific pathogen-free conditions at the Experimental Animal Center of the University of Science and Technology of China and used at the age of 6–12 weeks. All of experimental procedures involving animals were in accordance with the National Guidelines for Animal Usage in Research (China) and permission for these animal studies was obtained from the Ethics Committee at the University of Science & Technology of China.

### Cell isolation

To isolate hepatic lymphocytes, livers were passed through a 70 μM filter, resuspended in 40% Percoll diluted in PBS (GE Healthcare), gently overlaid onto 70% Percoll and centrifuged at 1,260 g for 30 min. Hepatic lymphocytes were then collected from the interlayer. To isolate uterine and SG lymphocytes, uterus or SG tissue samples were cut into pieces, digested with Collagenase IV (Sigma) in complete Iscove's Modified Dulbecco's Medium (IMDM), and lymphocytes were collected using 70% Percoll in the same manner as that described for liver. To isolate splenic cells, spleen tissue samples were passed through a 70 μM filter and erythrocytes were lysed.

### Generation of induced NK cells from BM

The generation of induced NK cells from BM samples occurs in four steps, as described below.

Step 1: Bone marrow cells were collected from female mice (at the age of 6–8 weeks) treated intraperitoneally with 5-fluorouracil (Sigma) at 200 mg/kg body weight for four days previously.
Step 2: After lysing erythrocytes, BM cells were cultured in 6-well plates at a concentration of 1 × 10^7^ cells/well in 2 ml of complete Iscove's Modified Dulbecco's Medium (IMDM) containing 10% fetal bovine serum, 25 mM HEPES, 2 mM L-glutamine, 1% penicillin and streptomycin) and a mixture of recombinant murine stem cell factor (SCF) (50 ng/ml), IL-6 (10 ng/ml), and IL-3 (3 ng/ml) (all from PeproTech). The cultures were re-fed with the same medium (no IL-3) on day 3. On day 6 the culture were harvested, washed and Lineage^+^ cells were then depleted using a lineage cell depletion kit (Miltenyi Biotec).
Step 3: Purified lineage^−^ cells were cultured in 6-well plates at a concentration of 5 × 10^5^ cells/well in 2 ml of complete IMDM containing SCF (20 ng/ml), Flt3L (20 ng/ml) and IL-7 (10 ng/ml). On day 9, the cultures were re-plated in 6-cm dishes and 2 ml of fresh complete IMDM with SCF (10 ng/ml), Flt3L (10 ng/ml), and IL-7 (5 ng/ml) was added.
Step 4: On day 12, the cultures were re-plated in 10-cm dishes and 4 ml of fresh IMDM and SCF (5 ng/ml), Flt3L (5 ng/ml), IL-15 (60 ng/ml), and IL-2 (400 U/ml) was added. Every 3–4 days, half of the media was gently removed from the cultures, and 4 ml of fresh medium with SCF (5 ng/ml), Flt3L (5 ng/ml), IL-15 (30 ng/ml), and IL-2 (200 U/ml) was added. The supernatant was mixed by gentle shaking. Following the generation of induced NK cells on day 26–33, the addition of SCF or Flt3L was terminated; this allowed cells to be passaged if required.

### Stimulation of induced NK cells

Induced NK cells were washed and cultured at a concentration of 5 × 10^5^ cells/ml in complete IMDM with IL-15 (30 ng/ml), IL-2 (200 U/ml), and either IL-4 or IL-4 + STAT6 inhibitor (AS 1517499, Axon Medchem) at different concentrations for 3 days.

### Flow cytometry

The antibodies used for flow cytometry analysis are purchased from BD Biosciences [mAb to CD45 [30-F11], mAb to CD19 [1D3], mAb to CD49a [Ha31/8], mAb to CD49b [DX5], mAb to CD69 [H1.2F3], mAb to CD62L [MEL-14], mAb to NKG2D [CX5], mAb to CD11b [M1/70], mAb to Ly49A [A1], mAb to PD-L1 [MIH5], mAb to IL-4Rα [mIL-4R-M1], mAb to IL-12Rβ1 [114], mAb to IL-2Rα [PC61], mAb to IL-21R [4A9], mAb to IL-23R [3C9], or mAb to CD16 [2.4G2]], or from Biolegend [mAb to CD3ε [145-2C11], mAb to CD19 [6D5], mAb to NK1.1 [PK136], mAb to NKp46 [29A1.4], mAb to CD27 [LG.3A10], mAb to CD127 [A7R34], mAb to CD90.2 [30-H12], mAb to T-bet [4-B10], mAb to CXCR3 [CXCR3-173], mAb to IFN-γ [XMG1.2], or from eBioscience [mAb to NKp46 [29A1.4], mAb to Eomes [Dan11mag], mAb to TRAIL [N2B2], mAb to NKG2A [16a11], mAb to KLRG1 [2F1], mAb to Ly49G2 [4D11], mAb to Ly49C/I/F/H [14B11], or mAb to IL-15Rα [DNT15Ra]]. Stained cells were analyzed on a FACSCalibur, an LSR II, or an LSRFortessa flow cytometry system (BD Biosciences). For surface staining, cells harvested from cultures, or single-cell suspensions from various organs, were incubated for 15 min with rat immunoglobulin for 20 min to block Fc receptors. Cells were then stained with appropriate antibodies and a fixable viability kit (Zombie NIR, BioLegend). For the intranuclear staining of transcription factors, cells were first stained for surface markers, fixed and then permeabilized with the Foxp3 staining buffer set (eBioscience). Cells then were resuspended with permeabilization buffer and stained with antibodies. For the intracellular staining of cytokines, cells were seeded in 24 well plates and stimulated with phorbol 12-myristate 13-acetate (PMA) (50 ng/ml, Sigma) and ionomycin (1 μg/ml, Calbiochem) in the presence of monensin (10 μg/mL, Sigma) for 4 h. Cells were then washed, stained, fixed and permeabilized using the same procedure as that described for the intracellular staining of transcription factors. For cell death assays, cells were incubated with 1 μg/ml of 7-aminoactinomycin D (7-AAD) (eBioscience) before being analyzed in a flow cytometer. Cells positive for 7-AAD were considered to be dead.

### Quantitative real-time polymerase chain reaction

Total RNA from cells harvested from cultures, or from various organs, was extracted using TRIzol reagent (Invitrogen) and cDNA was synthesized with an oligo dT primer (Invitrogen) and M-MLV reverse transcriptase (Invitrogen). RNA expression was then analyzed by quantitative real-time PCR with SYBR Premix Ex Taq (Tli RNaseH Plus, TaKaRa) and appropriate primers. The expression of target mRNA was normalized to the expression of the housekeeping gene *Hprt*.

### Enzyme-linked immunosorbent assays (ELISA)

Induced NK cells were seeded at a concentration of 1 × 10^6^/well in a 24-well plate. Cells were cultured in complete IMDM with IL-15 (30 ng/ml), IL-2 (200 U/ml) and with or without IL-4 (0.5 ng/ml) at 37°C for 3 days. granulocyte-macrophage colony stimulating factor (GM-CSF) or placenta growth factor (PGF) in the supernatant was determined using an ELISA Kit for GM-CSF or PGF (Cloud-Clone Corp).

### Flow-based killing assays

Flow-based killing assays were performed as previously described (20). Briefly, the target YAC-1 cells were labeled at 37°C for 15 min with 200 nM CFSE (Sigma). Labeled cells were then seeded at a concentration of 2 × 10^4^/well in 96-well round bottom plates with induced NK cells (at E:T ratios of 0:1, 0.1:1, 1:1, 5:1, and 10:1), with and without stimulation by IL-4, in complete IMDM for 4 h. Immediately prior to flow cytometry, cells from each well were incubated with 1 μg/ml of 7-AAD. Cells positive for both 7-AAD and CFSE were considered as lysed targets. The proportion (%) of specific lysis was calculated by using the following equation: 100% × (% sample lysis–% basal lysis)/(100–% basal lysis) in which basal lysis represented cell lysis in the absence of any effectors.

### *In vivo* treatment with IL-4

At the age of 9 weeks, female mice were injected intravenously with IL-4 (10 mg per mouse) or PBS. After 36 h, the mice were sacrificed for further analysis.

### Statistical analysis

Statistical analyses were performed using GraphPad Prism Software. Data were analyzed using unpaired two-tailed *t* tests or one-way analysis of variance (ANOVA) followed by the Holm-Sidak test. Data are presented as means ± standard error of the mean (SEM). Statistical significance is given hereafter as ^*^p < 0.05, ^**^p < 0.01 or ^***^p < 0.005.

## Results

### Generation of CD49a^+^ NK cells from bone marrow haematopoietic progenitors

To investigate the developmental conditions of CD49a^+^ NK cells, we established an *in vitro* system in which BM cells differentiated into NK1.1^+^CD49a^+^ cells upon culture in multiple cytokine cocktails without feeders.

**Figure 1:**
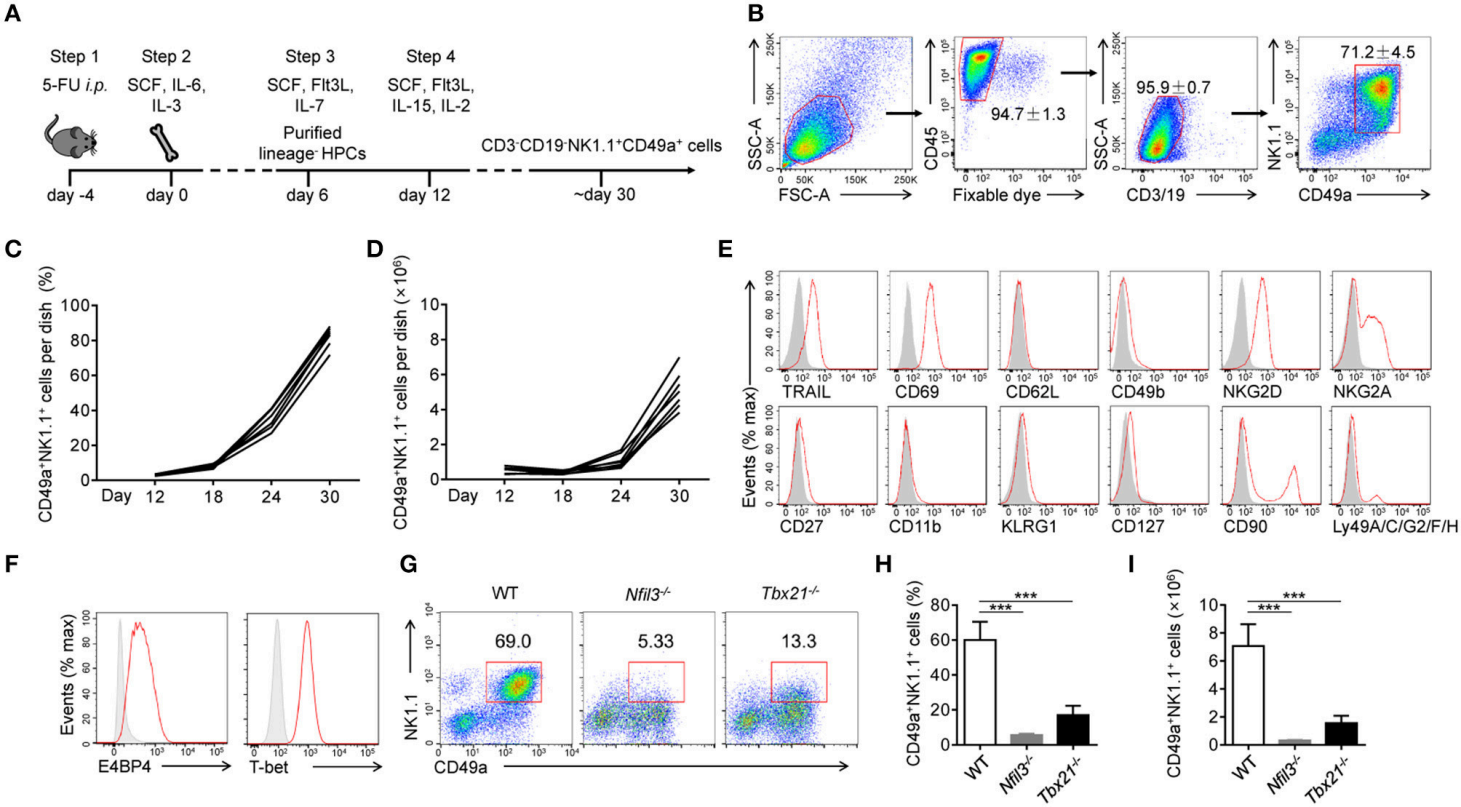
Generation and identification of CD49a^+^ NK cells. **(A)** Schematic of the procedure used to generate CD3^−^CD19^−^NK1.1^+^CD49a^+^ cells. **(B)** Gating strategy and representative flow plots of generated live CD45^+^CD3^−^CD19^−^NK1.1^+^CD49a^+^ cells. Numbers adjacent to the outlined areas indicate the proportion of cells (%), *n* = 8. **(C**,**D)** Flow cytometry analysis of frequency **(C)** and absolute number **(D)** for CD49a^+^ NK cells on day 12, 18, 24, and 30 in culture. Each line indicates cells in one of the culture dishes. *n* = 7. **(E)** Flow cytometry of the expression of various markers (horizontal axes, red histogram) compared with isotype control staining (gray histogram) in generated live CD45^+^CD3^−^CD19^−^NK1.1^+^CD49a^+^ cells on day 30. Data are representative of three independent experiments. **(F)** Flow cytometry of the expression of E4BP4 and T-bet (red histogram) compared with isotype control staining (gray histogram) in generated CD3^−^CD19^−^NK1.1^+^CD49a^+^ cells. Data are representative of three independent experiments. **(G,H)** Flow cytometry of cells generated from WT, *Nfil3*^−/−^ and *Tbx21*^−/−^ bone marrow cells on day 30 **(G)**. Numbers above the outlined areas indicate the proportion (%) of NK1.1^+^CD49a^+^ cells, gated on CD3^−^CD19^−^ cells. Frequency **(H)** and absolute number **(I)** of gated cells **(G)**. *n* = 4, 5, 4, respectively. Statistical analysis was performed by one-way analysis of variance (ANOVA) followed by the Holm-Sidak test. ***P < 0.001.

The generation of NK1.1^+^CD49a^+^ cells was recapitulated by a four-step process (Figure 1A). First (day−4-0), C57BL/6 WT mice were injected intraperitoneally with 5-fluorouracil to enrich hematopoietic progenitor cells (HPCs) (21). Second (day 0–6), BM cells were collected and cultured in Iscove's modified Dulbecco's medium (IMDM) containing stem cell factor (SCF), interleukin-6 (IL-6) and IL-3 to expand HPCs (22, 23). Third (day 7-12), purified lineage-negative (Lin^−^) HPCs were cultured with SCF, fms-like tyrosine kinase 3 ligand (Flt3L) and IL-7 (24). Fourth (day 12-), IL-15 and IL-2 were added to the culture and supplemented with low concentrations of SCF and Flt3L, to drive NK cell progenitors to differentiate into CD3^−^CD19^−^ NK1.1^+^CD49a^+^ cells (Figure 1B).

At the beginning of step 4 (day 12), NK1.1^+^CD49a^+^ cells were barely detectable in culture media. Afterwards, there was a notable increase in both the proportion and number of NK1.1^+^CD49a^+^ cells (Figures 1C,D). On day 30, and thereafter, a high proportion (70–95%) of NK1.1^+^CD49a^+^ cells were generated with a high proliferation rate (Figures 1C,D).

To investigate the lineage relationship of the NK1.1^+^CD49a^+^ cells generated, surface molecules were screened by flow cytometry (Figure 1E). Analysis showed that not only CD49a but also TRAIL and CD69 were expressed on the generated NK1.1^+^CD49a^+^ cells, but not CD62L and CD49b; collectively, these data were indicative of a tissue-resident phenotype. Generated NK1.1^+^ cells expressed NK-cell receptors such as NKG2D and NKG2A. Furthermore, the intracellular expression of E4BP4 and T-bet was detected in generated NK1.1^+^CD49a^+^ cells (Figure 1F). These are thought to be key transcription factors that regulate the development of NK cells from different organs (25, 26). To investigate the developmental dependency of these transcription factors, WT, *Nfil3*^−/−^, and *Tbx21*^−/−^ (T-bet-deficient) mice were injected with 5-fluorouracil and their BM cells collected to generate NK1.1^+^CD49a^+^ cells. It was found that very few NK1.1^+^CD49a^+^ cells developed from *Nfil3*^−/−^ BM cells, while some, but fewer cells, developed from *Tbx21*^−/−^ BM cells compared to the WT (Figures 1G–I). These results indicate that development of the generated NK1.1^+^CD49a^+^ cells was dependent on E4BP4 and partially dependent on T-bet; this scenario fits the dependence of NK cells.

To summarize, we successfully established a feeder-free system to generate NK1.1^+^CD49a^+^ cells from BM cells, and proved that these cytokine-induced cells are NK cells.

### Development and maintenance of generated CD49a^+^ NK cells require IL-15

**Figure 2:**
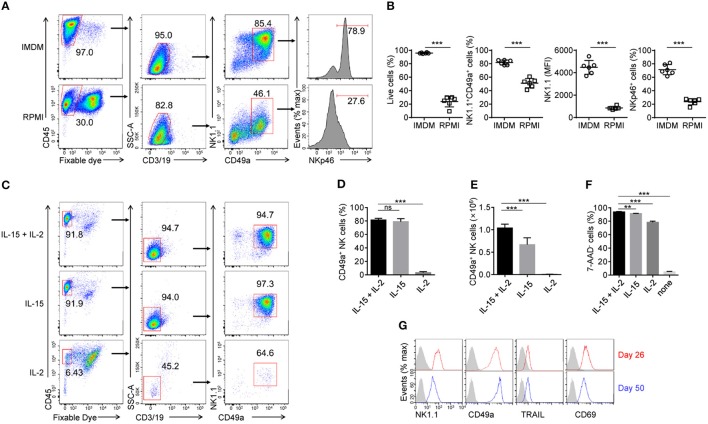
Effects of IL-15 and IL-2 upon the development and maintenance of generated CD49a^+^ NK cells. **(A)** Flow cytometry of generated cells cultured for 30 days in IMDM and RPMI medium. Numbers above the outlined areas and bracketed lines indicate the proportion (%) of cells. Data are representative of three independent experiments. **(B)** Frequency of live cells as a proportion of the number of total cells (far left), NK1.1^+^CD49a^+^ cells gated on live CD45^+^CD3^−^CD19^−^ cells (middle left), and frequency of NKp46^+^ cells (far right) and median fluorescence intensity (MFI) of NK1.1 (middle right) gated on live CD45^+^CD3^−^CD19^−^NK1.1^+^CD49a^+^ cells. Generated cells were cultured for 30 days in IMDM (circles) and RPMI (squares) medium. *n* = 8. Data are representative of three independent experiments. Statistical analysis was carried out using the unpaired *t* test. **(C)** Flow cytometry of generated cells in culture with IL-15 plus IL-2, IL-15 or IL-2. Numbers adjacent to the outlined areas and bracketed lines indicate the proportion (%) of cells. Data are representative of three independent experiments. **(D,E)** Frequency **(D)** and relative number **(E)** of generated CD49a^+^ NK cells in culture with IL-15 plus IL-2, IL-15 or IL-2. *n* = 4. Statistical analysis was carried out by one-way analysis of variance (ANOVA) followed by the Holm-Sidak test. **(F)** Frequency of 7-ADD^+^ induced NK cells cultured in fresh medium with IL-15 plus IL-2, IL-15, IL-2, or neither for 2 days. *n* = 4. Data are representative of three independent experiments. Statistical analysis was carried out by one-way analysis of variance (ANOVA) followed by the Holm-Sidak test. **(G)** Flow cytometry of the expression of NK1.1, CD49a, TRAIL, and CD69 on NK cells on day 50 (red histogram) compared with that on day 26 (blue histogram). The gray histogram indicates isotype control staining of induced NK cells. Data are representative of three independent experiments. **P < 0.01, ***P < 0.001.

Although widely used in cell culture, IMDM is less commonly used in the generation of murine NK cells compared with RPMI medium. We used IMDM to support the rapid proliferation of HPCs in step 2, as it contains more nutrients, such as glucose and amino acids, than RPMI medium. In order to choose a better medium for the generation of induced NK cells, purified Lin^−^ HPCs from step 2 were sequentially cultured in IMDM or RPMI media during steps 3 and 4. On day 30, we found that cells cultured in IMDM contained a higher proportion of live cells and induced NK cells with higher expression levels of NK1.1 and NKp46. These data indicate higher cell viability and increased induced NK cell generation and maturation compared with cells cultured in RPMI (Figures 2A,B). We therefore used IMDM throughout all subsequent culture experiments.

IL-15 is well known for its critical role in the development of NK cells from different organs, but not for all ILC1s (26, 27, 28, 29). We hypothesized that IL-15 is also the key factor for the generation of induced NK cells. To verify this, cultured cells from step 3 were sequentially cultured in the presence of IL-15, IL-2, or both, at the beginning of step 4 (day 18). Results showed that very few live induced NK cells were detected in the presence of IL-2 only, while there was no difference in the proportion of induced NK cells between cultures supplemented with IL-15 plus IL-2, and IL-15 only (Figures 2C–E). However, IL-2 increased the number of induced NK cells in the presence of IL-15 (Figure 2E). These results indicated that IL-15 is essential for the development of induced NK cells, while IL-2 only contributes to their proliferation.

To investigate whether IL-15 or IL-2 can maintain the survival of induced NK cells, the cells were washed twice with fresh IMDM, cultured for 2 days in fresh medium with IL-15, IL-2, both, or neither, then stained with 7-ADD and analyzed by flow cytometry. The results showed that the culture of induced NK cells without IL-15 and IL-2 quickly led to cell death, but only IL-15, or a low concentration (200 U/ml) of IL-2, could mainly ensure the maintenance of induced NK cells (Figure 2F). Even in cultures maintained for 50 days, induced NK cells still proliferated during step 4 (data not shown) and sustained their phenotypes in the presence of IL-15 and IL-2 (Figure 2G).

Thus, both the development and maintenance of induced NK cells require IL-15, while IL-2 contributes to their maintenance.

### Generated CD49a^+^Eomes^−^ NK cells share phenotypes with liver-resident NK cells

**Figure 3:**
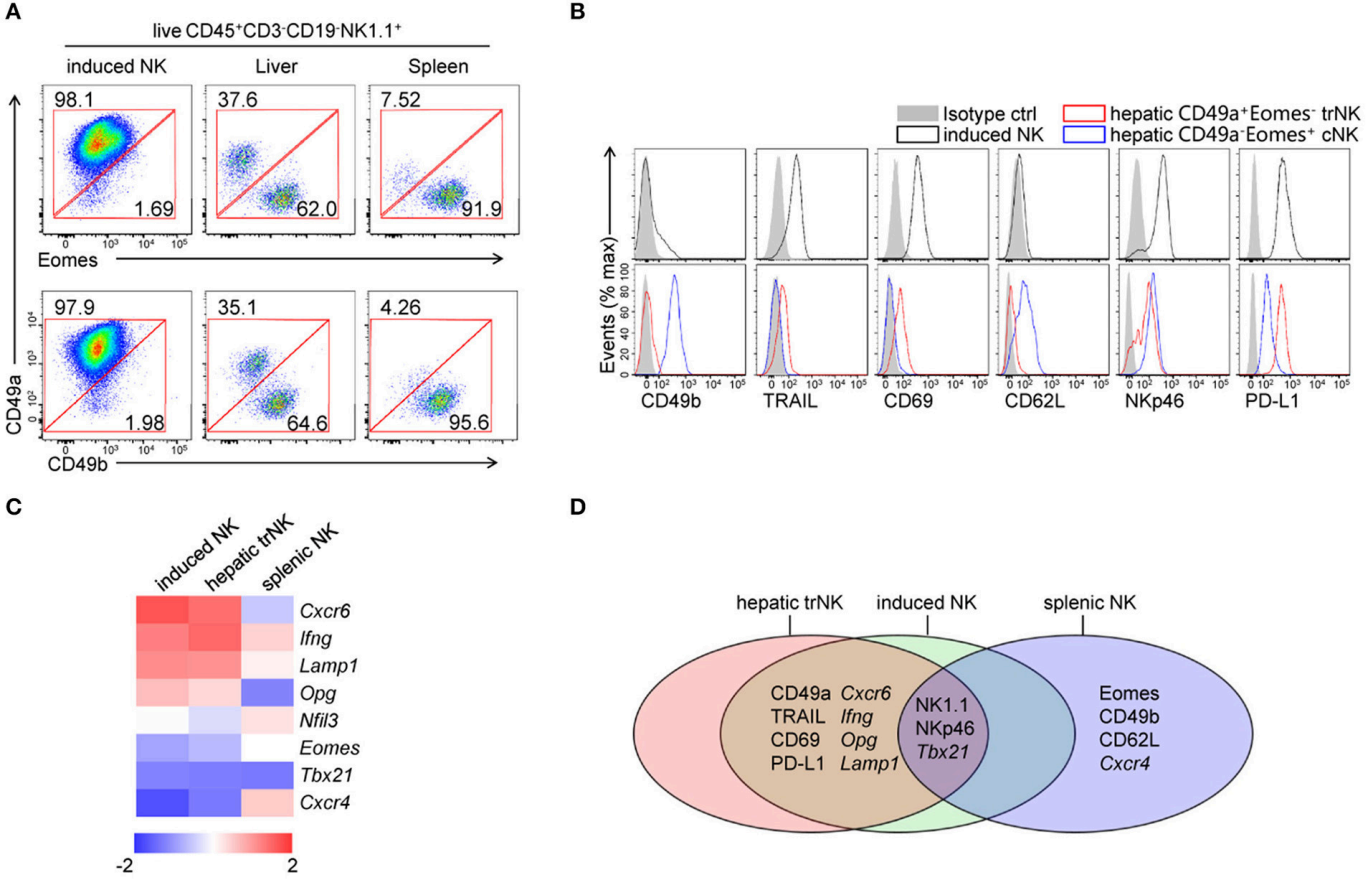
Comparisons of generated CD49a^+^Eomes^−^ NK cells, hepatic trNK cells and splenic cNK cells. **(A)** Flow cytometry of induced NK cells and cells isolated from the liver and spleen. Numbers adjacent to the outlined areas indicate the proportion (%) of cells, gated on live CD45^+^CD3^−^CD19^−^NK1.1^+^ cells. Data are representative of three independent experiments. **(B)** Flow cytometry of induced NK cells (live CD45^+^CD3^−^CD19^−^NK1.1^+^CD49a^+^, top) and hepatic NK subsets (live CD45^+^CD3^−^CD19^−^NK1.1^+^CD49a^+^Eomes^−^/CD49a^−^Eomes^+^, bottom), assessing the expression of CD49b, TRAIL, CD69, CD62L, NKp46, PD-L1 (open histogram) and isotype control staining (filled histogram). Data are representative of three independent experiments. **(C)** Heat map of genes related to transcript factors, cytokines and chemokine receptors in sorted induced NK cells, hepatic CD49a^+^ NK cells and splenic NK cells. Gene expression was analyzed by quantitative PCR and normalized to distribute from −2 to 2. Data are representative of three independent experiments. **(D)** Venn diagram depicting gene expression among induced NK cells, hepatic CD49a^+^ NK cells and splenic NK cells, as analyzed by quantitative PCR and/or flow cytometry.

To investigate the similarities between induced NK cells *in vitro* and tissue NK cells *in vivo*, we compared the expression of CD49a, Eomes and CD49b in induced NK cells, hepatic NK cells and splenic NK cells by flow cytometry (Figure 3A). Low levels of expression of Eomes and CD49b were detected in CD49a^+^ induced NK cells, which was similar to hepatic CD49a^+^ trNK cells. We further found that induced NK cells also shared similar expression levels of multiple surface molecules (CD49b, TRAIL, CD69, CD62L, NKp46, and PD-L1) with hepatic CD49a^+^Eomes^−^ trNK cells, but were distinct from CD49a^−^Eomes^+^ cNK cells (Figure 3B). Gene expression of chemokine receptors, transcription factors and cytokines was screened in sorted induced NK cells, hepatic trNK cells and splenic NK cells; results showed that a number of genes shared a similar expression level in induced NK cells and hepatic trNK cells (Figures 3C,D). Thus, generated CD49a^+^Eomes^−^ NK cells share similar phenotypes with liver-resident NK cells.

### IL-4 converts CD49a^+^Eomes^−^ NK cells to CD49a^+^Eomes^+^ NK cells

**Figure 4:**
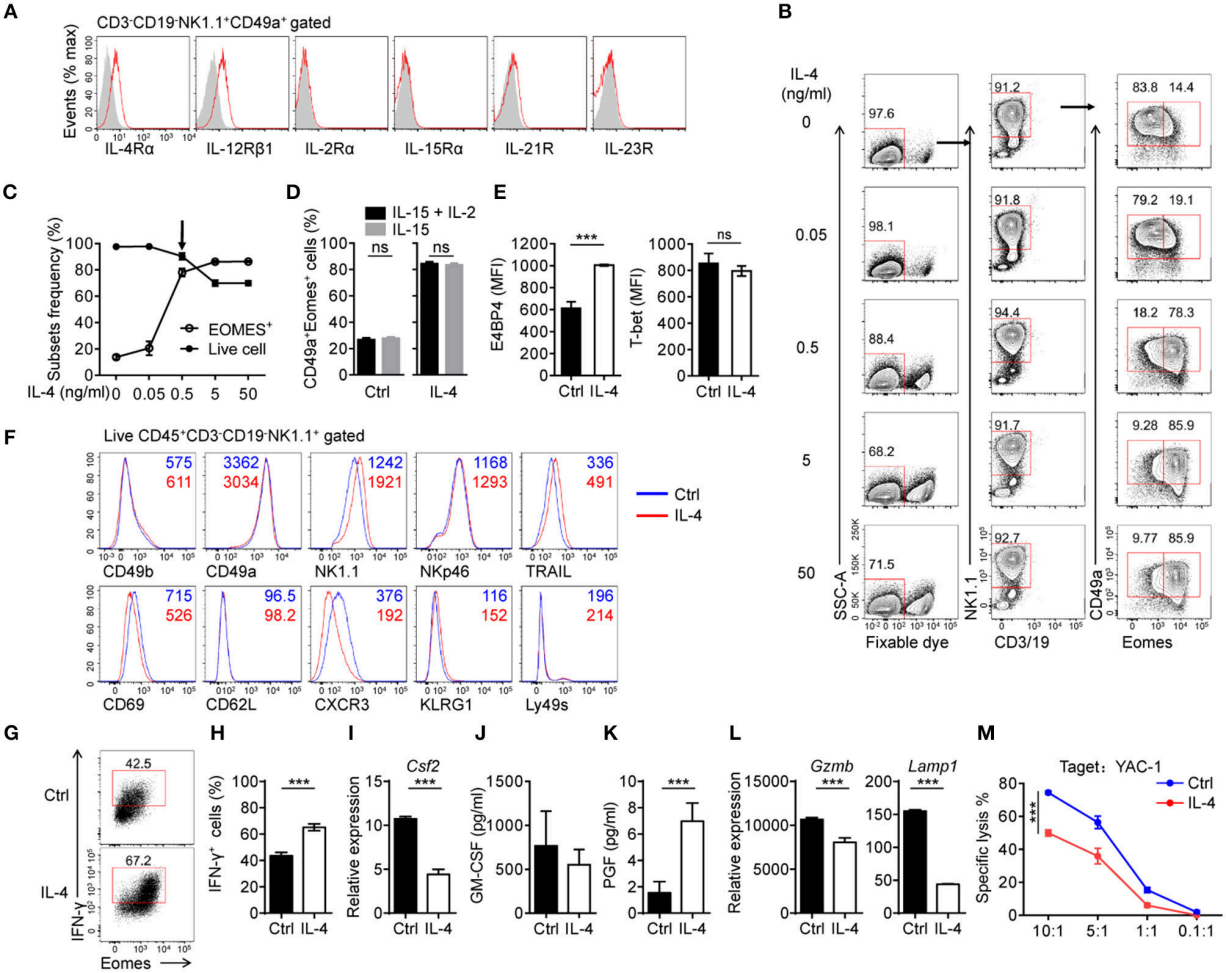
IL-4 converts generated CD49a^+^Eomes^−^ NK cells to CD49a^+^Eomes^+^ NK cells. **(A)** Flow cytometry of the expression of cytokine receptors (horizontal axes, red histogram) compared with isotype control staining (gray histogram) in generated live CD45^+^CD3^−^CD19^−^NK1.1^+^CD49a^+^ induced NK cells. Data are representative of three independent experiments. **(B)** Flow cytometry of induced NK cells stimulated for 3 days in medium containing IL-4 (0, 0.05, 0.5, 5, and 50 ng/mL) supplemented with IL-15 (30 ng/mL) and IL-2 (200 U/mL), assessing the frequency of live CD3^−^CD19^−^NK1.1^+^CD49a^+^Eomes^−/+^ cells. Numbers above the outlined areas indicate the proportion (%) of cells. *n* = 3 for each dose of IL-4. Data are representative of three independent experiments. **(C)** Frequency of live cells (filled symbol) and live CD3^−^CD19^−^NK1.1^+^CD49a^+^Eomes^+^ cells (open symbol). *n* = 4. **(D)** Frequency of CD49a^+^Eomes^+^ induced NK cells which were generated in culture with IL-15 plus IL-2 or IL-15, and stimulated with IL-4 (0.5 ng/mL) for 3 days. Cells were gated on live CD45^+^CD3^−^CD19^−^NK1.1^+^ cells. Data were analyzed statistically by the unpaired *t* test. **(E)** MFI of E4BP4 (left) and T-bet (right) in induced NK cells stimulated for 3 days with or without IL-4 (0.5 ng/mL). *n* = 4. Data are representative of three independent experiments. Data were analyzed statistically by the unpaired *t* test. **(F)** Flow cytometry of induced NK cells after 9 days stimulation with (red) or without (blue) IL-4 (0.5 ng/mL), assessing the expression of various markers (horizontal axes). Cells were gated on live CD45^+^CD3^−^CD19^−^NK1.1^+^ cells. Data are representative of three independent experiments. **(G,H)** Flow cytometry of the intracellular expression of IFN-γ by induced NK cells stimulated with or without IL-4 (0.5 ng/mL) for 3 days, and stimulated with PMA and ionomycin in the presence of monensin for 4 h. *n* = 4. Data are representative of three independent experiments. Data were analyzed statistically by the unpaired *t* test. **(I,L)** Quantitative PCR analysis of the expression of *Csf2* **(I)**, *Gzmb*, and *Lamp1* **(L)** from purified induced NK cells after 3 days in culture with or without IL-4 (0.5 ng/mL). Results are presented relative to the expression of *Hprt*. Data are representative of three independent experiments. Data were analyzed statistically by the unpaired *t* test. **(J,K)** Enzyme-linked immunosorbent assay for GM-CSF **(J)** and PGF (**K**) in the supernatants of cells from induced NK cells, stimulated with or without IL-4 (0.5 ng/mL) for 3 days. *n* = 4. Data were analyzed statistically by the unpaired *t* test. **(M)** Killing assay involving YAC-1 targets incubated for 4 h at the indicated effector:target ratios, with induced NK cells cultured with or without IL-4 (0.5 ng/mL) for 3 days. *n* = 4 for each effector:target ratio. Data were analyzed statistically by the unpaired *t* test. ***P < 0.001.

In addition to CD49a^+^Eomes^−^ trNK cells in liver, new subsets of trNK cells, expressing both CD49a and Eomes, have been reported in tissues such as the uterus and SG, however the development of these cells still remains unclear (13, 30). To explore the conditions in which induced NK cells can induce the expression of Eomes, we wondered if there were any other cytokines that cells would respond to in culture. Flow cytometry showed that IL-4Rα was expressed on induced NK cells, as well as IL-12Rβ1 (Figure 4A). IL-4 has been reported to promote the expression of Eomes by CD8^+^ T cells via both STAT6-dependent and independent ways (31, 32). We suspected that IL-4 could induce the expression of Eomes in induced NK cells. To verify this, induced NK cells were cultured in medium containing IL-4 at different concentrations supplemented with IL-15 and IL-2 for 3 days. Interestingly, an IL-4-dose-dependent increase of Eomes was detected by intracellular staining (Figures 4B,C). Even at a low concentration (0.5 ng/ml) of IL-4, most induced NK cells were converted to Eomes^+^ cells. It should be noted that significant cell death occurred when the level of IL-4 reached 5 ng/mL or more. Thus, 0.5 ng/ml was chosen as an optimal dose of IL-4 with which to stimulate induced NK cells.

It has been reported that IL-2 is essential for inducing and maintaining the expression of IL-4Rα during the differentiation of T helper type 2 cells (33). However, induced NK cells, which were generated and stimulated in the absence of IL-2, still responded to IL-4 and upregulated the expression of Eomes (Figure 4D).

We then found that E4BP4 was also upregulated upon stimulation of IL-4, and that levels of T-bet remained unchanged (Figure 4E). Next, we considered whether induced NK cells would change their phenotypes in response to IL-4. Induced NK cells were stimulated with IL-4 for 9 days with a half medium change every 3 days and analyzed the expression of surface molecules by flow cytometry (Figure 4F).

Most of the molecules tested showed little change in their expression, including tissue-resident markers (CD49b, CD49a, and CD69), with the exception of NK1.1 (increased) and CXCR3 (decreased). These data indicate that Eomes-expressing induced NK cells do not develop into cNK cells or lose markers associated with tissue residency.

Next, we tested whether IL-4 altered the functions of induced NK cells. The expression of IFN-γ by IL-4-stimulated induced NK cells was increased and most IFN-γ^+^ cells are Eomes^+^ cells (Figures 4G,H). We found that IL-4 suppressed the expression of granulocyte-macrophage colony-stimulating factor (GM-CSF) mRNA (*Csf2*) in induced NK cells, although these cells maintained a high level of GM-CSF secretion (Figures 4I,J). GM-CSF has been reported to be secreted by trNK cells in the uterus, where this cytokine has functions in implantation, invading placental morphogenesis, and the recruitment of immune cells (34). Furthermore, IL-4 promoted the secretion of placental growth factor (PGF) by induced NK cells (Figure 4K), which can be secreted by uterine NK cells and contributes to angiogenesis in the early decidua (35, 36). To assess the cytotoxicity of IL-4-stimulated induced NK cells, we first analyzed the expression of granzyme B (encoded by *Gzmb*) and CD107a (encoded by *Lamp1*) by quantitative PCR, and found that both of these genes were downregulated (Figure 4L). Subsequently, a killing assay against YAC-1 target cells showed a reduction of cytotoxicity in IL-4-stimulated induced NK cells (Figure 4M).

These results indicate that IL-4 converted CD49a^+^Eomes^−^ induced NK cells to CD49a^+^Eomes^+^ induced NK cells, and altered their function of cytokine secreting and less cytotoxicity.

### IL-4-induced CD49a^+^Eomes^+^ NK cells share phenotypes and functions with uterine NK1.1^+^CD49a^+^Eomes^+^ cells

**Figure 5:**
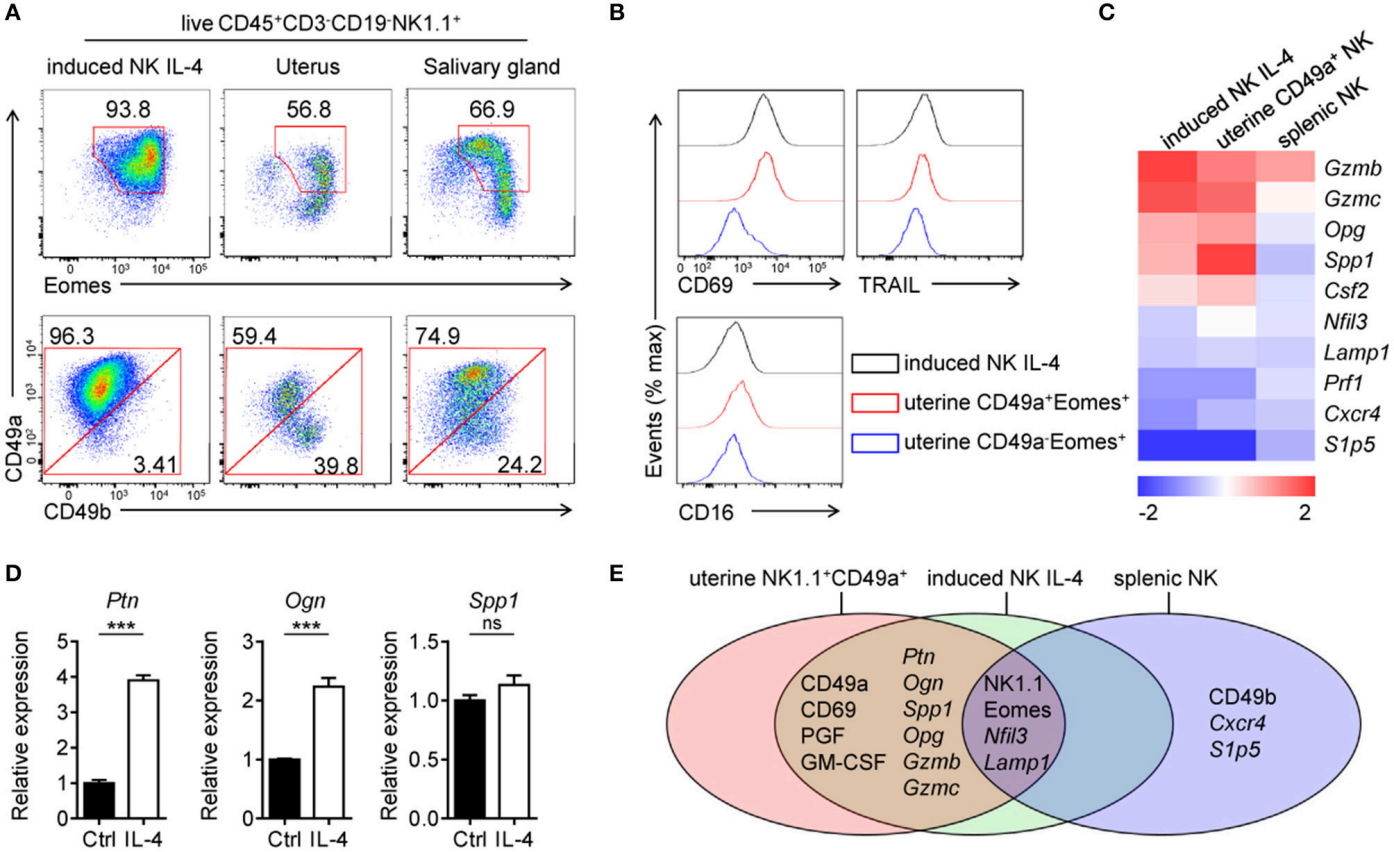
Comparisons of IL-4 stimulated CD49a^+^Eomes^+^ induced NK cells and uterine NK1.1^+^CD49a^+^Eomes^+^ cells. **(A)** Flow cytometry of induced NK cells stimulated with IL-4 and cells isolated from the uterus and salivary gland. Numbers adjacent to the outlined areas indicate the proportion (%) of cells, gated on live CD45^+^CD3^−^CD19^−^NK1.1^+^ cells. Data are representative of three independent experiments. **(B)** Flow cytometry of induced NK cells (CD45^+^CD3^−^CD19^−^NK1.1^+^CD49a^+^) stimulated with IL-4 and uterine NK1.1^+^ subsets (CD49a^+^Eomes^+^ and CD49a^−^Eomes^+^ cNK in CD45^+^CD3^−^CD19^−^NK1.1^+^), assessing the expression of CD69, TRAIL and CD16. **(C)** Heat map of genes related to transcription factors, cytokines and chemokine receptors in sorted induced NK cells, uterine NK1.1^+^CD49a^+^ cells and splenic NK cells. Gene expression was analyzed by quantitative PCR and normalized to distribute from −2 to 2. Data are representative of three independent experiments. **(D)** Quantitative PCR analysis of the expression of *Ptn, Ogn*, and *Spp1* from purified induced NK cells after 3 days in culture with or without IL-4 (0.5 ng/mL). Results are presented relative to the expression of *Hprt*. Data are representative of three independent experiments. Data were analyzed statistically by the unpaired *t* test. **(E)** Venn diagram depicting gene expression in induced NK cells after 3 days in culture with IL-4, uterine NK1.1^+^CD49a^+^ cells and splenic NK cells, analyzed by quantitative PCR and/or flow cytometry. ***P < 0.001.

After stimulation with IL-4, CD49a^+^ induced NK cells exhibit Eomes^+^ and uterine NK-like characteristics. IL-4-stimulated induced NK cells were then compared with CD3^−^CD19-NK1.1^+^ cells in the uterus as well as in SG (Figure 5A). The analysis of CD49a, Eomes and CD49b expression by flow cytometry revealed that IL-4-stimulated induced NK cells share similar primary phenotypes with NK1.1^+^CD49a^+^Eomes^+^CD49b^−^ uterine cells, while NK cells in SG expressed higher level of CD49b in CD49a^+^ cells and a higher level of CD49a expression in Eomes^+^ cells. In addition, IL-4-stimulated induced NK cell expressed the same levels of CD69, TRAIL, and CD16 as uterine NK1.1^+^CD49a^+^Eomes^+^ cells (Figure 5B). Gene expression screening showed that a number of genes shared the same expression level in IL-4-stimulated induced NK cells and uterine NK1.1^+^CD49a^+^Eomes^+^ cells (Figure 5C). Our previous work has reported that these induced uterine NK-like cells showed high expression levels of growth-promoting factors (GPFs), such as PTN, OGN and OPN. These GPFs were secreted from both human and murine decidual NK cells, and were shown to promote fetal growth (10). In addition, IL-4-stimulation enhanced GPF *Ptn* and *Ogn* gene expression in induced NK cells (Figure 5D). Through adoptive transfer, these induced NK cells were trafficked to the decidua and functioned to reverse impaired fetal growth in *Nfil3*^−/−^ mice and aged mice (10). Thus, it was concluded that IL-4-induced CD49a^+^Eomes^+^ NK cells shared both phenotypes and functions with NK1.1^+^CD49a^+^Eomes^+^ cells (Figure 5E).

### The IL-4/STAT6 axis is important for the generation of CD49a^+^Eomes^+^ induced NK cells

**Figure 6:**
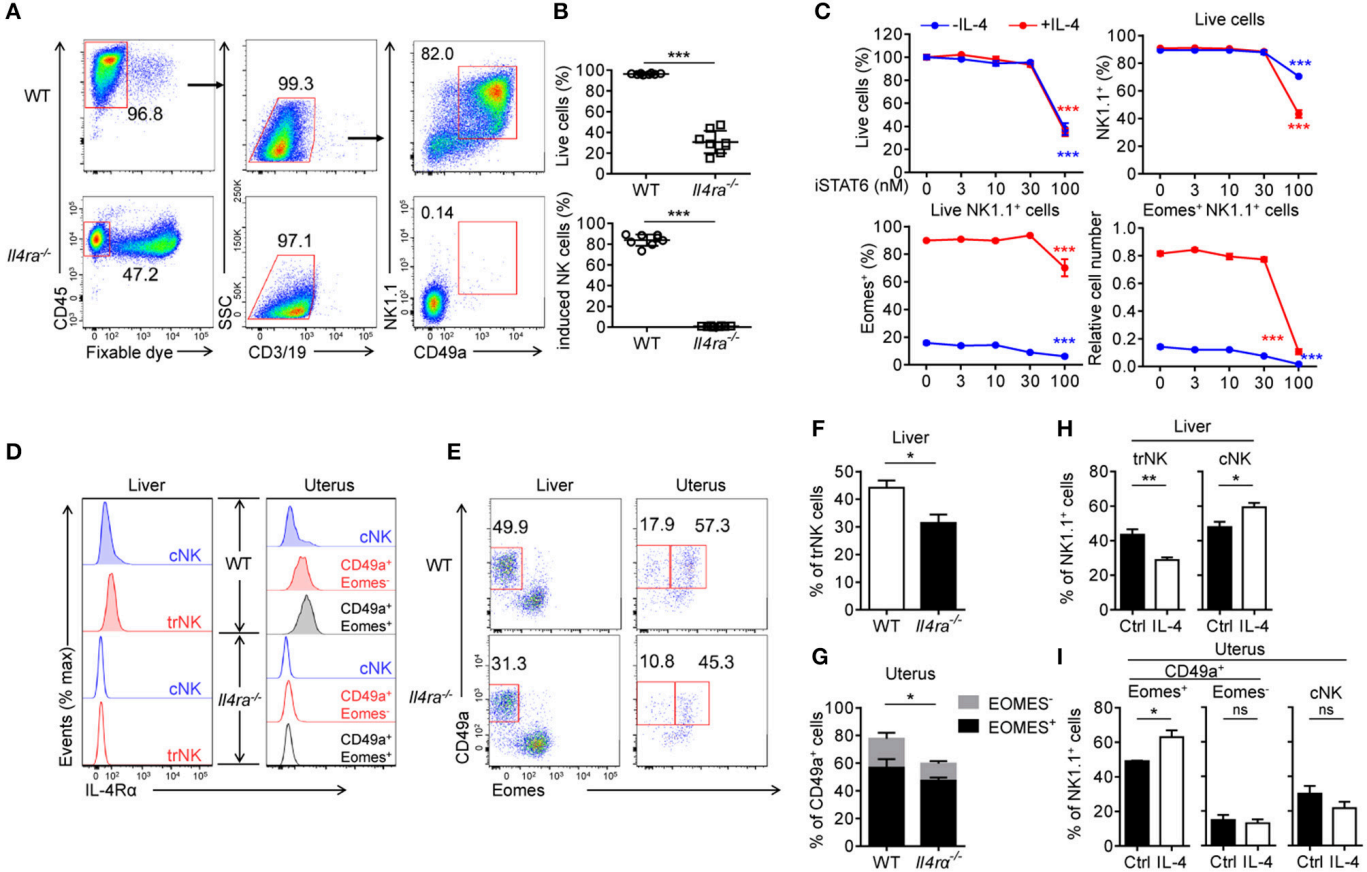
The IL-4/STAT6 axis is important for the generation of CD49a^+^Eomes^+^ induced NK cells. **s(A)** Flow cytometry of generated cells cultured for 30 days from WT or *Il4ra*^−/−^ BM. Numbers adjacent to the outlined areas indicate the proportion (%) of cells. **(B)** Frequency of live cells in total cells (top) and CD49a^+^ induced NK cells in live CD45^+^CD3^−^CD19^−^ cells (bottom), generated from WT (circles) or *Il4ra*^−/−^ (squares) BM. *n* = 8. Data were analyzed statistically by the unpaired *t* test. **(C)** Frequency of live cells as a proportion of the total number of cells (top left), NK1.1^+^ cells in live cells (top right), Eomes^+^ in live NK1.1^+^ cells (bottom left) and cell number of live Eomes^+^NK1.1^+^ cells (bottom right) relative to the original cell number. Generated cells were stimulated for 3 days with STAT6 inhibitor (0, 3, 10, 30, and 100 nM), supplemented with (red) or without (blue) IL-4 (0.5 ng/mL). *n* = 4 for each condition. Data are representative of three independent experiments. Data from induced NK cells stimulated with 0 and 100 nM STAT6 inhibitor were compared, and statistical analysis was performed by one-way analysis of variance (ANOVA) followed by the Holm-Sidak test. **(D)** Flow cytometry of IL-4Rα on NK1.1^+^ cell subsets from the liver and uterus of WT (filled) and *Il4ra*^−/−^ (open) mice. Data are representative of three independent experiments. **(E)** Flow cytometry of live CD45^+^CD3^−^CD19^−^NK1.1^+^ cells from the liver and uterus of WT and *Il4ra*^−/−^ mice. Numbers above the outlined areas indicate percent hepatic trNK cells, uterine CD49a^+^Eomes^−^ (left) and CD49a^+^Eomes^+^ cells (right). Data are representative of three independent experiments. **(F)** Frequency of trNK cells from the liver of WT and *Il4ra*^−/−^ mice. n = 4, 5. Data were analyzed statistically using the unpaired *t* test. **(G)** Frequency of CD49a^+^Eomes^+^ cells and CD49a^+^Eomes^−^ from the uterus of WT and *Il4ra*^−/−^ mice. *n* = 4, 5. Frequencies of CD49a^+^ cells (Eomes^+^ and Eomes^−^) were analyzed statistically using the unpaired *t* test. **(H,I)** Frequency of CD45^+^CD3^−^CD19^−^NK1.1^+^ cell subsets from the liver **(H)** and uterus **(I)** of WT mice injected intravenously with IL-4 (10 mg per mouse) or PBS (ctrl) 36 h before harvest. *n* = 6 for liver and *n* = 3 for each 2 uterus. Data were analyzed statistically by the unpaired *t* test. *P < 0.05, **P < 0.01, ***P < 0.001.

To further investigate the role of IL-4 in the development of CD49a^+^ NK cells, we created IL-4Rα deficient (*Il4ra*^−/−^) mice, as described in previous reports (10, 37, 38). Surprisingly, these mice failed to generate induced NK cells from BM cells (Figures 6A,B). This result indicated that IL-4 not only induces Eomes; it is also necessary for the generation of CD49a^+^Eomes^−^ induced NK cells. As STAT6 is activated downstream of IL-4, we considered whether IL-4 regulates the generation and expression of Eomes in induced NK cells via STAT6. An inhibitor of STAT6 (AS 1517499) was added to culture medium containing induced NK cells with or without IL-4. After 3 days, it was evident that STAT6 inhibition led directly to cell death and the downregulation of both NK1.1 and Eomes. As a result, the number of Eomes^+^ induced NK cells decreased, even in the presence of IL-4 (Figure 6C). Thus, STAT6 plays important roles in the survival of induced NK cells and the expression of Eomes by stimulation of IL-4. To confirm whether IL-4 signaling participates in the development of trNK cells *in vivo*, we analyzed the expression of IL-4Rα on CD3^−^CD19^−^NK1.1^+^ cells from both liver and uterus. Compared with the *Il4ra*^−/−^ mice, cNK cells from WT liver and uterus expressed low levels of IL-4Rα, while trNK cells and ILC1s expressed higher levels than cNK cells. Next, we observed a significant reduction in the proportion of trNK cells in *Il4ra*^−/−^ liver and NK1.1^+^CD49a^+^ cells in *Il4ra*^−/−^ uterus, compared with WT mice (Figures 6D–G). On the other hand, an intravenous injection of IL-4 led to a reduction in the proportion of trNK cells in the liver but an increase proportion of NK1.1^+^CD49a^+^Eomes^+^ cells in the uterus (Figures 6H,I). No CD49a^+^Eomes^+^ NK cells in the liver of IL-4-injected mice were detected. In summary, the IL-4/STAT6 axis plays an important role in the generation of CD49a^+^Eomes^+^ induced NK cells.

## Discussion

In this study, we constructed an *in vitro* system which could be used to generate CD49a^+^Eomes^−/+^ induced NK cells and found that the development and maintenance of these cells required IL-15. We also found that IL-4 can induce the expression of Eomes in induced NK cells, and that Eomes^+^ induced NK cells share both phenotypes and functions with uterine NK1.1^+^CD49a^+^Eomes^+^ cells.

It has been reported that progenitors from BM or fetal liver develop into hepatic trNK cells as well as all type of ILCs when cultured with feeders *in vitro* (18, 19). In our system, BM progenitors are committed to differentiate into CD49a^+^ NK cells without feeders. This system has significant potential for both developmental and functional research in CD49a^+^Eomes^−/+^ subsets of NK cells, and provides a more appropriate method for transfer. Furthermore, the migratory potential and fetal-growth-promoting effect of induced NK cells clearly indicates their potential for adoptive NK cell transfer therapies.

BM cells from *Nfil3*^−/−^ mice failed to generate induced NK cells, while the proportion of NK1.1^+^CD49a^+^ cells were significantly reduced, but still present, in their livers and uterus (8, 30, 39), as well as *Il4r*α^−/−^ mice. This suggests that subsets of NK1.1^+^CD49a^+^ cells derived from different progenitors would differ in terms of their relative dependencies (30, 40). Moreover, *Nfil3*^−/−^ mice showed impaired pregnancy and fetal growth (8, 17). Our study therefore provides clues as to the key factors involved in the development of functional uterine NK1.1^+^CD49a^+^ cells.

Progenitors from BM have been reported as a source of hepatic trNK cells and uterine NK cells *in vivo* (26, 41). The data presented herein support these earlier findings in that the generation of CD49a^+^ NK cells from BM progenitors share the phenotype, gene expression, and function with both hepatic and uterine NK1.1^+^CD49a^+^ cells. Recent studies have shown that cNK cells can be converted into cells with ILC1 phenotypes when cultured with transforming growth factor-β (TGF-β). In addition, TGF-β signaling is thought to be critical for the development of SG NK1.1^+^CD49a^+^ cells (3) as well as the conversion of cNK cells into ILC1s in the tumor microenvironment (42). The Kopcow group previously reported that when cultured in the combined conditions of hypoxia, TGF-β1, and a demethylating agent, NK cells from human peripheral blood obtain decidual NK cell markers and functions (43, 44). However, we did not apply TGF-β in our system, and very few CD49a^−^CD49b^+^ NK cells were detected in culture; these cells were also Eomes^−^. Thus, we consider that CD49a^+^ induced NK cells possibly develop directly from progenitors in our system without conversion from CD49a^−^CD49b^+^Eomes^+^ NK cells.

We also found that IMDM is more suitable for the generation of CD49a^+^ NK cells than RPMI medium. The concentration of aromatic amino acids (L-Tyrosine, L-Phenylalanine, and L-Tryptophan) in IMDM is 3–5 fold higher than in RPMI, and these amino acids provide the source of AhR ligands to promote the differentiation of Th17 (45). It has also been reported that the maintenance of hepatic trNK cells require AhR (46), and that *Ahr*^−/−^ uterine NK cells lose their ability to trigger spiral arterial remodeling (47). Thus, it is speculated that the aromatic amino acids in IMDM could also support the differentiation of CD49a^+^ NK cells. However, in order to fully confirm this, further investigation is needed.

The Eomes transcription factor has been reported to be a basic requirement for the development of mature cNK cells (48). The data presented herein show that IL-4 induced the expression of Eomes in generated CD49a^+^ NK cells. Moreover, we found that low concentrations of IL-4 could promote the expression of cytokines, and suppress the cytotoxicity of induced NK cells, which were phenotypically and functionally similar to uterine NK1.1^+^CD49a^+^ cells. It should be noted that NK1.1^+^CD49a^+^ cells, especially in the uterus, expressed higher levels of IL-4Rα than cNK cells, and that additional IL-4 supplemented *in vivo*, increased the proportion of uterine NK1.1^+^CD49a^+^Eomes^+^ cells. On the other hand, although there was a reduced proportion of NK cells, NK1.1^+^CD49a^+^ NK cells are still present in *Il4ra*^−/−^ mice, suggesting that other compensatory mechanisms may be involved in the development of uterine NK1.1^+^CD49a^+^ cells, or that only some of the subsets of cells are affected by IL-4 signaling. It is somewhat surprising that the number of hepatic trNK cells reduced after injection with IL-4. One possible supposition is the loss of homeostasis or tissue residency in such cells when the expression of Eomes in hepatic CD49a^+^ NK cells was up-regulated by stimulation with IL-4 (15).Thus, these findings suggest a possible developmental mechanism for NK1.1^+^CD49a^+^ cells.

This study also links IL-4 with uterine NK cells, as well as pregnancy. IL-4 is detectable at the feto–maternal interface (49) and a decrease or lack of IL-4 can lead to a series of pregnancy disorders (50, 51). The Du group previously reported that the IL-4-STAT6 pathway promotes the expression of Tim-3 on human peripheral NK cells, which mediate immunoregulation in maternal-fetal immune tolerance (52, 53). The absence of IL-4 does not affect the generation and homeostasis of cNK cells (54), but can induce the *in vivo* production of IFN-γ by NK cells (55). The Miyajima group previously showed that in mice overexpressing IL-4, NK cells change their phenotypes, produce higher levels of IFN-γ, IL-10, and GM-CSF, and increase cytotoxicity compared with cNK cells in control mice (56). These observations provide evidence of a cytokine-promoting effect for IL-4, both *in vivo* (55) and *in vitro* (57), but also that the opposite effect is true for cytotoxicity *in vitro* (58). The Miyajima group did not detect changes in trNK cell subsets from different organs in IL-4-overexpressing mice. In Miyajima's report, as the concentration of IL-4 in serum reached 100 ng/ml (56), there was most likely a dose-and site-dependent difference operating in terms of the stimulation of NK cells by IL-4.

## Ethics statement

All animal studies were approved by the ethics committee of the University of Science and Technology of China(USTCACUC1601005). The sample size was determined by the resource equation method.

## Author contributions

XN designed and conducted experiments, analyzed data, and wrote the manuscript. JZ created the *Il4ra*^−/−^ mice. RS established the FACS techniques and interpreted the data. BF and ZT provided strategic planning, conceived the project, and interpreted some of the data. HW designed the research, supervised the work and revised the manuscript.

### Conflict of interest statement

The authors declare that the research was conducted in the absence of any commercial or financial relationships that could be construed as a potential conflict of interest.
